# Prickly Pear By-Product in the Feeding of Livestock Ruminants: Preliminary Investigation

**DOI:** 10.3390/ani10060949

**Published:** 2020-05-30

**Authors:** Massimo Todaro, Marco Alabiso, Antonino Di Grigoli, Maria Luisa Scatassa, Cinzia Cardamone, Isabella Mancuso, Francesca Mazza, Adriana Bonanno

**Affiliations:** 1 Department of Agricultural, Food and Forest Sciences (SAAF), University of Palermo, viale delle Scienze n. 13, 90128 Palermo, Italy; marco.alabiso@unipa.it (M.A.); antonino.digrigoli@unipa.it (A.D.G.); francesca.mazza@unipa.it (F.M.); adriana.bonanno@unipa.it (A.B.); 2 Istituto Zooprofilattico Sperimentale della Sicilia (IZS), via G. Marinuzzi, 3, 90129 Palermo, Italy; luisa.scatassa@izssicilia.it (M.L.S.); cinzia.cardamone@izssicilia.it (C.C.); isabella.mancuso@izssicilia.it (I.M.)

**Keywords:** prickly pear by-product, chemical composition, storage

## Abstract

**Simple Summary:**

The question of sustainability of animal nutrition has become a popular topic. The gains made by recycling safe, yet otherwise valueless, by-products and wastes from human food and fiber production (green feeds) may lessen the competition between humans and animals for crops and decrease the environmental impact of food production. In this context, prickly pear by-product, which results from *Opuntia ficus-indica* (L.) Mill. fruits processed for juice extraction, could be an optimum by-product to ruminant feeding. This study evaluates the nutritional characteristics and its stability during storage using potassium metabisulfite as a preservative agent. This preliminary investigation showed this by-product could represent, for its chemical composition, an interesting and suitable feeding source to be used to increase the environmental and economic sustainability of ruminant livestock. On the basis of the results, the use of preservative was able to slightly slow down the early phase of the acidification process and limit the presence of spoilage microorganisms after a three-week storage period. The high content of soluble sugars in the prickly pear by-product suggests that a likely storage technique could be ensiling the mass with straw.

**Abstract:**

In Sicily, the current increasing cultivation of *Opuntia ficus-indica* corresponds to an availability of prickly pear by-product (PPB) that results from fruit processing for juice extraction. This investigation aims to evaluate the nutritional traits of PPB for ruminant feeding and its stability during a 21-day outdoor storage, using potassium metabisulfite (PMB) as a preservative agent, added to the PPB mass at different doses (0, 50, 100, and 150 g/kg). The fractioning of PPB showed that it included 28% of peel and pulp and 72% of seeds on a dry matter (DM) basis. On the whole, this by-product was low in crude protein (5.32% DM), high in fiber content (51.38%, 41.15% and 14.64% DM for NDFom, ADFom and ADL respectively), non-fiber carbohydrates (NFC, 29.68% DM), and soluble sugars (13.3% DM), with a moderate level of net energy for lactation (4.59 MJ/kg DM). Storage was the main factor of alteration of PPB chemical composition with the exception of ether extract. A decline of NFC and soluble sugars, due to microbial fermentation, was observed with all PMB treatments, especially during the first week of storage, probably due to evolution of both coccus (M17) and rod LAB (MRS), which increased their loads at the seventh day of storage.

## 1. Introduction

The Cactaceae family, with about 1600 species, is cultivated worldwide for fruits, forage, fodder, and even as a vegetable. Cacti are well adapted to arid and semi-arid regions where food and fodder crops are limited, and are naturalized in several areas over the world, including the Mediterranean basin, Middle East, South Africa, Australia, and India [1]. 

Among many species of the Cactaceae producing edible fruits, those of the genus *Opuntia* have the most relevant role in agriculture. In particular, *Opuntia ficus-indica* (L.) Mill, commonly known as prickly pear, is cultivated for fruit production on all continents except Antarctica. The prickly pear produces sweet, nutritionally rich edible fruits, and its tender cladodes are used as a fresh green vegetable and salad [2]. In some countries, different parts of the plant are utilized in both food and cosmetic industries [3]. A growing interest in *Opuntia* spp. crops is also related to the use of its biomass for biofuel production [4,5,6]. Several publications from the United Nation’s Food and Agricultural Organization (FAO) summarize investigations into localization and use of *Opuntia* as forage resource and its feeding quality for livestock [7,8,9]. In this regard, prickly pear provides an excellent and cheap alternative feed to supplement livestock diets [10,11,12,13] due to its efficiency in converting water to dry matter (DM.) In fact, during periods of drought, ranchers allow cattle and sheep to eat the pads, after burning off the spines, as a resource of both food and water [14,15,16,17]. When sheep fed with spineless cactus exceeded 300 g/d dry matter, their drinking water intake approached zero [18,19].The more recent studies investigating the effects of *Opuntia* forage on rumen digestion, weight gain and other physiological traits in cattle, sheep, or goats showed that the animals fed diets in which *Opuntia* forage was supplemented with protein-rich feeds improved their health status and reproductive performance [20]. However, the use of *Opuntia* in combination with other feed sources can be opportune to also reduce forage crop demand for ruminants in dry-land areas [20]. Moreover, the prickly pear cladodes resulted in being highly palatable and digestible for both wild and domesticated rabbits [21]. 

Indeed, prickly pear represents a digestible energy-balanced feed [22] being moderately high in sugars, starch, ether extract, crude protein, amino acids, and fiber [1,23], and provides the animals’ requirements for vitamins and calcium [24]. 

With regard to prickly pear fruit, it shows high levels of betalains, total carotenoids, ß-carotene, ascorbic acid, and is one of the best sources in total phenolic compounds [25,26]. The nutritional and health benefits of prickly pear fruit are related to its antioxidant properties due to ascorbic acid, polyphenols, especially flavonoid compounds (e.g., kaempferol, quercetin, and isorhamnetin), and the mixture of yellow betaxanthin and red betacyanin pigments [27]. Meanwhile, the free radical scavenging activity of the red cactus pears was related to the concentration of total phenolic compounds and ascorbic acid [28].

In Sicily (Italy), the current growing cultivation of *Opuntia ficus-indica* corresponds to an increasing availability of a prickly pear by-product (PPB) in which residues from fruits processed for juice extraction, comprising of peel, pulp, and seeds. This by-product, available only recently in a large amount, is little known and studied, but could be a very interesting source for ruminant nutrition.

Actually, in livestock farms, the PPB is stored only by keeping it in uncovered heaps placed outdoors, and gradually administered to the animals in this fresh status. However, the high sugar content of the PPB quickly grafts bacterial fermentations, requiring the use of a fermentative stabilizer. For this purpose, a natural stabilizer, such as potassium metabisulfite (PMB), could be effective to control the growth of spoilage microorganisms [29]. Thus, PPB was added with different doses of potassium metabisulfite (50 g, 100 g, and 150 g/100 kg PPB), established to remain strongly below the LD_50_ (lethal dosage to kill 50% of a population of test animals, that is 2 g/kg of body weight), and to have a very low risk to reach the maximum amount recommended by WHO for human consumers (0.7 mg/kg of body weight).

The aim of this preliminary study was to evaluate the nutritional characteristics of PPB for ruminants feeding and its stability during a 21-day outdoor storage with increasing amounts of potassium metabisulfite to prevent spoilage.

## 2. Material and Methods

At the end of August, an amount of 80 kg of PPB was taken directly from the prickly pear juice extraction industry, in the province of Palermo in Sicily, and transferred in the experimental laboratories of Department of Agricultural, Food, and Forest Science at the University of Palermo. After arrival, a sample was taken for pH measurement, chemical analysis, and evaluation of seeds, pulp, and peel proportions.

The 80 kg of PPB was partitioned into four parts of 20 kg, and each part was treated with a different PMB doses, as follows: T0, control, without PMB; T50, with PMB addition at dose of 50 g/100 kg of PPB; T100, with PMB addition at dose of 100 g/100 kg of PPB; T150, with PMB addition at dose of 150/100 kg of PPB. The powdered PMB was well mixed to allow a uniform distribution in the whole mass. Then each treated PPB mass was divided into two heaps, used as replicates. The eight uncovered heaps of 10 kg were placed outdoors on a breathable plastic sheet, simulating the usual storage technique of this by-product in livestock farms. 

Samples of PPB were taken from each uncovered heap at days 1, 3, 7, 14, and 21 to measure pH and determine the chemical composition, and at days 1, 7, and 21 to determine the microbiological profile. Measurements of pH in PPB samples were performed directly with the HI 9025 142 pH meter equipped with a spear electrode FC 200 (Hanna Instruments Inc., Woonsocket, RI, USA).

### 2.1. Chemical Parameters

The PPB samples were analysed according to the procedures of the AOAC [30] to determine dry matter (DM, 934.01), ether extract (EE, 920.39), crude protein (CP, 2001.11), and ash (942.05). The fiber fractions NDF (NDFom, 2002.04), ADF (ADFom, 973.18) and ADL (973.18) were determined in accordance with AOAC [30] and Van Soest et al. [31], and expressed exclusive of residual ash. Non-fiber carbohydrate (NFC) content was calculated as (100 − [CP + EE + ash + NDFom]). The net energy for lactation (NE_L_, MJ/kg DM) of feeds was estimated using relationships from the Institut National de la Recherche Agronomique (INRA) system [32]. 

The soluble sugars analysis was performed by colorimetric estimation following the anthrone method [33] and using a HACH DR/4000U spectrophotometer (HACH, Loveland, CO, USA) to read the absorbance at 640 nm.

### 2.2. Microbiological Analysis

A total of 30 g of each PPB sample was weighed into sterile stomacher bags and homogenized with 270 mL Saline Peptone Solution in a stomacher (Type 400; Seward London, UK). Decimal dilutions were prepared with the same diluent and 1 mL of each suspensions were plated and incubated as follows:

- Total mesophilic microorganisms on plate count agar (PCA), incubated aerobically at 30 °C for 72 h [34];
- Enterobacteriaceae on Violet Red bile glucose agar (VRBGA) incubated aerobically for 24 h at 37 °C [35];
- Coliforms on Violet Red bile agar (VRBA) incubated at 37 °C for 24 h [36];
- *Escherichia coli* β-glucuronidase positive on Tryptone Bile X-Gluc agar (TBX) incubated aerobically for 24 h at 44 °C [37];
- Coagulase-positive *Staphylococci* on Baird Parker with RPF supplement, incubated aerobically for 24–48 h at 37 °C [38];
- Sulfite reducing anaerobes on Iron sulfite agar (ISA) incubated at 37 °C for 24 h [39];
- Molds on Dichloran Rose Bengal chloramphenicol agar (DRBC) incubated aerobically for five days at 25 °C.

Detection of *Salmonella* spp. and *L. monocytogenes* was carried out by an enzyme-linked fluorescent assay (ELFA) in an automatic system VIDAS (bioMerieux, Marcy-l’Etoile, France). In particular, 25 g of each sample were weighed into sterile stomacher bags and homogenized with 225 mL of enrichment broth for each pathogen:

- Detection of *Salmonella* spp. consisted in enrichment buffered peptone water (BPW) with *Salmonella* supplement incubated at 41.5 °C for 18–24 h and a subsequent step performed by VIDAS UP (SPT).
- Detection of *L. monocytogenes* consisted in enrichment in LMX broth with LMX supplement incubated at 37 °C for 26–30 h and then a subsequent step performed by VIDAS *L. monocytogenes* Xpress (LMX).

Enumeration of lactic acid bacteria (LAB) was carried out as follows: mesophilic and thermophilic rod-shaped LAB on de Man-Rogosa-Sharpe (MRS) agar acidified with 5 M lactic acid to pH 5.4 incubated anaerobically at 30 and 44 °C for 48 h, respectively, using the AnaeroGen AN25 (Oxoid, Milan, Italy) in jars closed hermetically; mesophilic and thermophilic coccus-shaped LAB on Medium 17 (M17) agar incubated aerobically for 48 h at 30 and 44 °C, respectively [40].

### 2.3. Statistical Analysis

Statistical analysis was carried out using the GLM procedure in SAS 9.2 software [41]. In the ANOVA model, the treatment with PMB (T0, T50, T100, and T150) and storage time (1, 3, 7, 14, and 21 days for chemical parameters, and 1, 7, and 21 days form microbiological data) were fixed factors; the interaction between treatment and storage was also considered, and the related means were reported in the tables only when such interaction was significant. When a significant effect (*p* < 0.05) was detected, Tukey’s test was used for means comparisons. Before statistical analysis, microbiological loads were transformed into logarithmic form (log_10_).

## 3. Results and Discussion

The fractioning of PPB showed that it included 28% of peel and pulp (PP) and 72% of seeds on a DM basis. The seed composition was higher in ether extract and NDF, and comparable for DM and crude protein than those of PP and PPB. On the whole, this new by-product resulted in lower moisture and protein contents (Table 1) if compared to another fresh by-product available in Sicily for ruminant feeding, such as fresh lemon pulp, in which Todaro et al. [42] detected levels of 19.2% and 11.5% for DM and protein, respectively. Instead, the fiber fractions of PPB showed higher values than those observed in the fresh lemon by-product, which exhibited 39.0%, 23.9% and 7.7% of NDF, ADF, and ADL, respectively [42]; these differences can be attributed to the presence of pectin, which determines an overestimation of the ADFom content and, consequently, an underestimation of hemicelluloses, and can be related also to the very high presence of seeds, which characterizes this particular fruit and its by-product, and contributes to increasing its lignin content. However, Ozcan and Al Juhaimi [43] report that fatty acids composition of prickly pear seeds is characterized by 61% linoleic acid and 25% oleic acid. Due to their high fiber content, the net energy for lactation of PPB and its components showed modest values, especially if compared to those of citrus by-products reported by Bampidis and Robinson [44], ranging from 7.2 to 7.7 MJ/kg DM, probably due to the higher lignin content of PPB. The pH value of fresh PPB resulted quite comparable than that of other fresh citrus by-products [44]. 

In Sicily, the availability of PPB occurs in a period of poor natural feeding resources, so this product rich in water and digestible fiber can represent an important dietary component for the nutrition of small ruminants. Commonly, the farming use of PPB implies that the fresh by-product is stored in heaps outdoors until it is entirely consumed; in these conditions, it is necessary to evaluate its stability during storage, from both a chemical and a microbiological point of view. Table 2 reports the variations of PPB chemical composition during 21 days of storage, in relation to increasing levels of PMB utilized to modulate fermentative processes.

The statistical analysis showed that the storage time was the main factor of alteration of PPB chemical composition. Indeed, during storage, all chemical components exhibited significant modifications, with the exception of ether extract. On the whole, the storage time was responsible for the decline in NFC and soluble sugars, principally due to fermentation of sugars, which corresponded to an increasing trend recorded for DM, crude protein, fiber fractions, and ash. It can be noted that the decline involving NFC and sugars occurred quickly during the first week of storage and then it stopped; at the same time, the pH values, after a gradual reduction during the first week, probably as a consequence of the production of lactic acid from sugars fermentation, showed a substantial rise at day 21, presumably due to a deacidification following the use of acid lactic for microbial growth. Moreover, protein, NFC, soluble sugars, and pH were significantly affected by the interaction between PBM treatment and storage time. The least square means of these interactions, are reported in Table 2. The increase of protein over time was less consistent in the treatments with 100 and 150 g/kg of PMB, and at 21 days of storage the higher and lower protein levels were recorded without any treatment and using the maximum PMB dose, respectively. Moreover, the NFC decline occurred until day 7 with all PMB treatments. Nevertheless, at the third day the NFC values of samples treated with 100 and 150 g/kg of PMB were significantly higher (*p* < 0.05) than the T0 and T50 theses, indicating a probable effect of the highest PMB doses in slowing the initial phase of fermentation; however, such treatments lost this their effect already at day 7. An analogous trend was observed on soluble sugars (Table 2), confirming that the highest PMB doses (100 and 150 g/kg), utilized to preserve the product, had a certain efficacy in modulating the fermentation only for the first three days of storage. On the basis of these trends of fermentative processes, the decline of pH until day 7 of storage, and its subsequent raise occurred with all PMB treatments. Additionally, for this parameter, significant differences (*p* < 0.05), following the use of different PMB doses, were observed on the third day of sampling, when T100 and T150 thesis showed higher pH values than T0 and T50, in line with the trend recorded for NFC and soluble sugars. Moreover, at 21st day, the pH value recorded without any PMB treatment resulted statistically higher (*p* < 0.01) than those of the other PPB heaps treated with different PMB doses, indicating a greater deacidification. This latter result can be explained by the reduction of lactic acid, used to support a more rapid microbial growth favored in the untreated PPB heaps, and also proved by the high protein level detected with T0 at 21 days, since microbes are mainly composed of protein, as suggested by Wizna et al. [45]. 

The reduction of the sugar content and pH value in the PPB samples during the first week of storage seems to be linked to the development of the microorganisms active in the sugar fermentation (Table 3). This relationship was confirmed by the evolution of both coccus (M17) and rod LAB (MRS), responsible of fermentation processes, which increased their loads at day 7 of storage, and then slightly decreased. Additionally, the total mesophilic count (PCA) showed an increase up to the seventh day with all treatments but, successively, its load increased again with T0 and T50 theses, and remained stable with the highest PMB doses, determining the significance of the interaction. Indeed, at 21st day, higher bacterial loads were found in correspondence to lower PMB doses (T0 and T50) (*p* < 0.01). The interaction between PMB treatment and storage time affected also the loads of coliforms (VRBA) and *Enterobacteriaceae* (VRBGA), which, at the seventh day, were found only in the untreated samples, confirming the efficacy of PMB in the control of the growth of spoilage microorganisms, as reported by Rana et al. ([29]. However, at the 21st day, VRBA and VRBGA were found again in all samples, although with lower loads for PPB samples treated with 100 and 150 g/kg of PMB, indicating an effect of the higher PMB doses in restraining the coliforms and *Enterobacteriaceae* development after a three-week storage. Accordingly, the lowering of coliforms and *Enterobacteriaceae*, together with that of total mesophilic bacteria, detected at 21 days using the maximum PMB dose can be considered responsible of the reduction of PPB protein level. Other spoilage microorganisms, as *Escherichia coli*, coagulase-positive *Staphylococci*, sulfite-reducing anaerobes and molds were not found in PPB samples because they were below the detection limit; moreover, pathogenic microorganisms, as *Listeria monocytogenes* and *Salmonella* spp. were not detected.

These preliminary results seem to be promising. However, the conservation method operated by farmers needs to be improved, in order to stabilize the mass and preserve the nutritional value and safety of this by-product. Thus, further investigations are required to check and set up other and effective methods to preserve the PPB safety during storage; in this regard, the high content of soluble sugars in the PPB suggests that a likely storage technique could be ensiling the mass with straw, also considering that PPB and wheat straw are available contemporarily at the end of summer. Moreover, other studies may be made on this feeding resource in order to evaluate its antioxidant properties derived from its high polyphenol content that can be beneficial to livestock animals and consumers of their products. However, it is also appropriate to evaluate the daily dose of use of PPB, since by-products from fruit processing with high polyphenol content may contain anti-nutritional factors [46] able to modulate the rumen microbiota composition by negatively affecting some species of fibrolytic bacteria and ciliate protozoa [47]. 

## 4. Conclusions

This preliminary investigation showed as PPB could represent, for its chemical composition, an interesting and suitable feeding source to be used to increase the environmental and economic sustainability of ruminant livestock. On the basis of the results, the use of PMB as a preservative agent added to the PPB mass at doses of 100 and 150 g/kg was able to slightly slower down the early phase of the acidification process and limit the presence of coliforms and *Enterobacteriaceae* after a three-week storage period. Further investigations have to be performed to improve the method to preserve the PPB mass during storage, and verify the production responses in ruminant species. 

## Figures and Tables

**Table 1:** Chemical composition of prickly pear by-product (PPB) and its fractions.

Items	PPB	Peel and Pulp (28%)	Seeds (72%)
Dry matter (%)	30.45	30.87	30.56
Crude protein (%DM) ^1^	5.32	5.00	5.75
Ether extract (%DM)	5.04	3.60	8.54
NDFom (%DM) ^2^	51.38	41.50	73.27
ADFom (%DM) ^3^	41.15	34.50	57.25
ADL (%DM) ^4^	14.64	5.13	21.37
NFC (%DM) ^5^	29.68	30.41	9.18
Ash (%DM)	8.58	19.49	3.26
NE_L_ (MJ/kg DM) ^6^	4.59	5.04	4.41
pH	5.00	5.01	5.02

^1^ DM: dry matter. ^2^ NDFom: neutral detergent fiber assayed with a heat stable amylase and expressed exclusive of residual ash. ^3^ ADFom: acid detergent fiber expressed exclusive of residual ash. ^4^ ADL: acid detergent lignin. ^5^ NFC: non-fiber carbohydrates = 100 − (CP + ether extract + ash + NDFom). ^6^ NE_L_: net energy for milk production according to INRA (2018).

**Table 2:** Chemical composition of prickly pear by-product (PPB) during storage and in relation to treatments with potassium metabisulfite (PMB) (least square means).

Items	^1^ PMB Treatment		Storage (Days)					SEM ^6^	*p*-Value		
			1	3	7	14	21		Treatment (T)	Storage (S)	T × S
Dry matter (DM)		%	30.05 AC	29.14 BC	28.36 BC	31.52 A	32.26 A	0.608	**	**	ns
Crude protein		% DM	5.32	6.06	6.65	7.02	7.45	0.258	*	***	*
	T0		5.32 ^A^	6.33 ^AC^	7.47 ^B^_x_	6.80 ^BC^	8.21 ^B^_x_				
	T50		5.32 ^A^	6.95 ^B^	6.52 ^B^_y_	7.86 ^C^	7.48 ^C^_x_				
	T100		5.32 ^A^	5.13 ^A^	6.51 ^B^_y_	6.59 ^B^	7.65 ^C^_x_				
	T150		5.32 ^A^	5.84 ^AC^	6.10 ^BC^_y_	6.84 ^B^	6.47 ^B^_y_				
Ether extract		% DM	5.04	4.60	5.08	4.29	4.63	0.262	ns	ns	ns
NDFom ^2^		% DM	51.38 ^B^	58.48 ^BC^	65.60 ^A^	64.68 ^A^	62.03 ^AC^	1.256	ns	***	ns
ADFom ^3^		% DM	41.15 ^A^	47.86 ^B^	50.63 ^B^	48.84 ^B^	48.91 ^B^	0.844	ns	***	ns
ADL ^4^		% DM	14.64 ^A^	15.93 ^AC^	17.89 ^B^	17.18 ^BC^	18.05 ^B^	0.511	ns	**	ns
Ash		% DM	7.81 ^a^	8.83 ^a^	8.86 ^a^	9.15 ^ab^	10.08 ^b^	0.941	ns	*	ns
NFC ^5^		% DM	30.45	22.03	13.82	14.85	15.80	0.413	ns	***	**
	T0		30.45 ^A^	20.96 ^B^_x_	15.53 ^C^	11.45 ^C^	15.34 ^C^				
	T50		30.45 ^A^	21.66 ^B^_x_	13.65 ^C^	14.96 ^C^	13.94 ^C^				
	T100		30.45 ^A^	24.55 ^B^_y_	10.64 ^C^	16.11 ^C^	15.91 ^C^				
	T150		30.45 ^A^	25.96 ^B^_y_	15.44 ^C^	16.89 ^C^	18.01 ^C^				
Soluble sugars		% DM	13.34	16.82	9.66	9.52	8.91	0.906	ns	***	**
	T0		13.34 ^A^	12.86 ^B^_x_	10.11 ^C^	10.08 ^C^	9.10 ^C^				
	T50		13.34 ^A^	14.29 ^B^_x_	9.33 ^C^	9.29 ^C^	9.22 ^C^				
	T100		13.34 ^A^	20.98 ^B^_y_	9.54 ^C^	9.37 ^C^	8.62 ^C^				
	T150		13.34 ^A^	19.15 ^B^_y_	9.67 ^C^	9.37 ^C^	8.68 ^C^				
pH			5.01	4.27	4.07	4.70	6.23	0.209	ns	***	***
	T0		5.01 ^A^	3.86 ^B^_x_	3.80 ^B^	5.02 ^A^	6.90 ^C^_X_				
	T50		5.01 ^A^	4.05 ^B^_x_	4.05 ^B^	4.38 ^B^	5.97 ^C^_Y_				
	T100		5.01 ^A^	4.42 ^B^_y_	4.21 ^B^	4.55 ^B^	5.67 ^C^_Y_				
	T150		5.01 ^Aa^	4.73 ^Ab^_z_	4.23 ^B^	4.84 ^Ab^	5.97 ^C^_Y_				

^1^ T0, T50, T100, and T150: treated with 0, 50, 100, and 150 g PBM per 100 kg of PPB. ^2^ NDFom: neutral detergent fiber assayed with a heat stable amylase and expressed exclusive of residual ash. ^3^ ADFom: acid detergent fiber expressed exclusive of residual ash. ^4^ ADL: acid detergent lignin. ^5^ NFC: non-fiber carbohydrates: 100 − (CP + ether extract + ash + NDFom). ^6^ SEM: standard error of mean. *** *p* ≤ 0.001; ** *p* ≤ 0.01; * *p* ≤ 0.05; ns: *p* > 0.05. On the row, values with different superscript letters are significant A,B,C: *p* ≤ 0.01; a,b: *p* < 0.05. On the column, values with different subscript letters are significant X,Y,Z: *p* ≤ 0.01; x,y,z: *p* ≤ 0.05.

**Table 3:** Microbiological evolution of prickly pear by-product (PPB) during storage and in relation to treatments with potassium metabisulfite (PMB) (Least Square Means).

Items	^1^ PMB Treatment		Storage (Days)			SEM ^7^	*p*-Value		
			1	7	21		Treatment (T)	Storage (S)	T × S
M17 30°C ^2^		Log CFU/g	7.15 ^a^	7.97 ^b^	6.91 ^a^	0.237	ns	*	ns
M17 44°C ^2^		Log CFU/g	5.60 ^A^	7.69 ^B^	6.01 ^A^	0.395	ns	**	ns
MRS 30°C ^3^		Log CFU/g	7.23 ^A^	8.12 ^B^	5.92 ^C^	0.173	ns	***	ns
MRS 44°C ^3^		Log CFU/g	5.52 ^A^	8.05 ^B^	5.07 ^C^	0.118	ns	***	ns
PCA ^4^		Log CFU/g	5.23	7.30	6.24	0.344	ns	**	**
	T0		5.23 ^Aa^	6.20 ^Ab^	8.30 ^B^_X_				
	T50		5.23 ^Aa^	6.26 ^Ab^	8.30 ^B^_X_				
	T100		5.23 ^a^	6.26 ^b^	6.64 ^b^_Y_				
	T150		5.23 ^a^	6.26 ^b^	5.97 ^b^_Y_				
VRBGA ^5^		Log CFU/g	5.11	4.21	4.81	0.661	ns	*	*
	T0		5.11 ^a^	5.40 ^a^_x_	5.62 ^a^_x_				
	T50		5.11 ^a^	<1.00 ^b^_y_	5.48 ^a^_x_				
	T100		5.11 ^a^	<1.00 ^b^_y_	3.18 ^c^_y_				
	T150		5.11 ^a^	<1.00 ^b^_y_	2.54 ^c^_y_				
VRBA ^6^		Log CFU/g	5.08	3.74	4.18	0.574	ns	*	*
	T0		5.08 ^a^	4.52 ^a^_x_	5.08 ^a^_x_				
	T50		5.08 ^a^	<1.00 ^b^_y_	5.08 ^a^_x_				
	T100		5.08 ^a^	<1.00 ^b^_y_	2.67 ^c^_y_				
	T150		5.08 ^a^	<1.00 ^b^_y_	2.15 ^c^_y_				

^1^ T0, T50, T100, and T150: treated with 0, 50, 100, and 150 g PBM per 100 kg of PPB. ^2^ M17, agar for coccus lactic acid bacteria (LAB); ^3^ MRS, de Man–Rogosa–Sharpe agar for rod LAB; ^4^ Total mesophilic microorganisms on plate count agar (PCA); ^5^ Violet Red bile glucose agar for Enterobacteriaceae (VRBGA); ^6^ Coliforms on Violet Red bile agar (VRBA); ^7^ SEM: standard error of mean. *** *p* ≤ 0.001; ** *p* ≤ 0.01; * *p* ≤ 0.05; ns: *p* > 0.05. On the row, values with different superscript letters are significant A,B,C: *p* ≤ 0.01; a,b,c: *p* ≤ 0.05. On the column, values with different subscript letters are significant X,Y: *p* ≤ 0.01; x,y: *p* ≤ 0.05.
